# Phenolic Acids-Rich Fractions from *Agaricus bitorguis* (Quél.) Sacc. Chaidam ZJU-CDMA-12 Mycelia Modulate Hypoxic Stress on Hypoxia-Damaged PC12 Cells

**DOI:** 10.3390/molecules25204845

**Published:** 2020-10-21

**Authors:** Hongyun Lu, Zhihua Jiao, Yingchun Jiao, Wei Wang, Qihe Chen

**Affiliations:** 1 Department of Food Science and Nutrition, Zhejiang University, Hangzhou 310058, China; luhongyun@zju.edu.cn; 2 Department of Cell Biology, School of Medicine, Johns Hopkins University, Baltimore, MD 21205, USA; zjiao3@jhu.edu; 3 Agriculture and Animal Husbandry College, Qinghai University, Xining 810016, China; jiaoyingchun@qhu.edu.cn; 4 Institute of Quality and Standard for Agriculture Products, Zhejiang Academy of Agricultural Sciences, Hangzhou 310021, China; 13738132996@126.com

**Keywords:** intracellular phenolic acids, *Agaricus bitorquis* (QuéL.) Sacc. Chaidam, structure characterization, anti-hypoxia activity

## Abstract

Hypoxia is a common pathological process in various clinical diseases. However, there is still a lack of effective anti-hypoxia active substances. *Agaricus bitorguis* (Quél.) Sacc Chaidam (ABSC) is a rare wild edible macrofungus that grows underground at high altitudes. Herein, intracellular phenolic acids-rich fractions (IPA) were extracted from ABSC ZJU-CDMA-12, and the structural characterization and anti-hypoxia activity of IPA on PC12 cells were elucidated as well. The results of HPLC-Q-TOF-MS illustrated that five kinds of IPA were isolated from ABSC, including (−)-epicatechin gallate, arabelline, yunnaneic acid D, 2′-*O*-p-hydroxybenzoyl-6′-*O*-trans-caffeoylgardoside,4′-*O*-methylgallocatechin-(4->8)-4′-*O*-methylepigallocatechin. IPA extracted from ABSC proved to show anti-hypoxia activity on hypoxia-damaged PC12 cells. Hypoxia enhanced reactive oxygen species (ROS) generation and reduced the mitochondrial membrane potential (ΔΨm) in PC12 cells, resulting in the inhibition of survival and induction of apoptosis in PC12 cells. Measurements of 100 μg/mL and 250 μg/mL IPA could significantly reduce hypoxia-induced damage in PC12 cells by decreasing overproduced intracellular ROS, improving ΔΨm, and reducing cell apoptosis rate. Our findings indicated that the IPA from ABSC potentially could be used as novel bioactive components applied to anti-hypoxia functional foods or medicines.

## 1. Introduction

Phenolic acids, one of the main classes of polyphenols, are one of popular and effective health-promoting compounds, which are proved to exhibit different pharmacological activities, such as anti-proliferation activities, antioxidant activity, anti-hyperlipidemia activity, and anti-inflammatory activity [1,2,3]. Moreover, they can significantly inhibit intracellular ROS generation, reduce the percentage of apoptotic cells, and decrease oxidative stress damage [4,5]. Notably, edible macrofungi have been identified as a source of phenolic acids with diverse beneficial properties [6,7,8].

*Agaricus bitorquis* (Quél.) Sacc. Chaidam (ABSC), a specific wild macrofungus in Qaidam Basin (Qinghai province, China), has been consumed as a locally traditional and popular edible and medicinal macrofungus for its plentiful nutrition and bioactive compounds. Many investigations have proved that extracts from medicinal macrofungi (such as *Ganoderma lucidum*, *Antrodia camphorata*, *Dictyophora indusiata*) are abundant sources of compounds with significant bioactive activities. Ganoderic acid (GA) extracted from *Ganoderma lucidum* was proved to suppress the proliferation of a highly metastatic lung cancer cell line 95-D in xenograft tumor-bearing nude BALB/c mice [9]. Bach et al. [10] reported that phenolics from edible mushrooms (such as *Agaricus bisporus*, *Flammulina velutipes*, *Lentinula edodes*, and *A. brasiliensis*) showed the antioxidant and antimicrobial activities in vitro, and the extracts from *A. brasiliensis* mushroom showed stronger antioxidant activities among them. Ethanolic extracts from *Pleurotus eryngii* fruiting bodies are reported to have anti-inflammatory properties by downregulating lipopolysaccharide-stimulated nitric oxide, prostaglandin E2, inducible nitric oxide synthase, and cyclooxygenase-2 expression in RAW264.7 macrophages [11].

Hypoxia is defined as a decrease in available oxygen reaching the tissues of the body, and it could lead to stress responses—including quicker respiratory rate, faster heartbeat, and higher blood pressure—to satisfy the oxygen delivery to tissues [12]. Hypoxic stress, a common concern in medicine and biology, can induce excess production of ROS to cause cellular injury and death [13]. The body function impairment induced by hypoxia was reported to be linked with the acute mountain sickness, cardiovascular disease, stroke, neurodegenerative diseases, and even death in many countries [14]. Chronic exposure to hypoxia is reportedly involved in defective vessel formation and maybe could limited O_2_ availability to the brain, causing neurological disabilities. Previously, phenolic acids have been proved to show the antioxidant and antiradical activities. Recently, researchers have demonstrated that phenolic acids have other bioactive effects. Hao et al. [15] reported that hydroxycinnamic acid extracted from corncob showed neuroprotective effects by inhibit Aβ40 fibrillation and attenuate Aβ40-induced cytotoxicity. Hua et al. [16] found that chicoric acid could improve heart and blood responses to hypobaric hypoxia in Tibetan yaks. Mishra et al. [17] demonstrated that phenolic rich fractions from mycelium and fruiting body of *Ganoderma lucidum* could inhibit bacterial pathogens mediated by generation of ROS and protein leakage and modulate hypoxic stress in HEK 293 cell line.

In our previous work, we demonstrated that polysaccharides isolated from ABSC showed anti-fatigue and anti-hypoxia activities [18,19,20], and abundant IPA were also detected in its secondary metabolites derived from cultured mycelia. However, the structure characterization and biological activities of IPA have been insufficiently investigated and their therapeutic potential has not yet been fully explored. Thus, in this study the IPA extracts were analyzed by HPLC-Q-TOF-MS to determine its possible phenolic compositions, and the anti-hypoxia activity of IPA extracts was investigated. This work provides useful information for the large-scale production of bioactive compounds from ABSC and could promote the application and commercial value of such special mushroom. 

## 2. Results

### 2.1. Compound Separation and Identification

**Figure 1:**
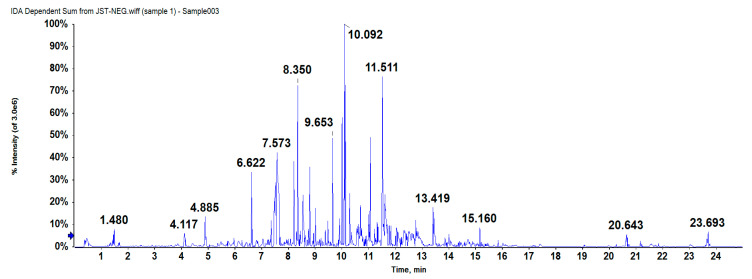
The chromatogram of IPA isolated from ABSC.

As can be seen in the base peak chromatogram (BPC) (Figure 1), 101 peaks were detected in IPA (75% ethanol extract) using a HPLC-Q-TOF-MS platform (data shown in Table S1). The identification of each peak was achieved according to its retention time, mass spectra, the information previously reported in literatures. Each peak was then elucidated in accordance with the reference HPLC chromatogram of standard solutions and related data. Nonetheless, only five phenolic compounds could tentatively be identified. Data obtained from HPLC-Q-TOF-MS of each compound (the retention time, detected and expected *m*/*z*, error (ppm), molecular formula, and fragments) are provided in Table 1, and Figure S1 shows the characteristic ion mass spectrometries of each compound.

Regarding the presence of phenolic acids and derivatives, five compounds including several isomers were detected. Arabelline was firstly detected in the species *Agaricus bitorquis*, and peaks at 7.475 min exhibited a quasimolecular ion at *m*/*z* 673.1774 with molecular formula C_32_H_34_O_16_ was expected to be Arabelline. It gave product ions at *m*/*z* 441.0845 corresponded to the losses of C_22_H_18_O_10_, possibly (−)-epicatechin gallate (ECG) derivative [21]. Besides, peaks at 8.827 min with molecular formula of C_32_H_32_O_15_ with ion (*m*/*z* 657.1778), produced a fragment ion at *m*/*z* 455.0980/561.1363/579.1468 has previously been described as 2′-*O*-*p*-hydroxybenzoyl-6′-*O*-trans-caffeoylgardoside [22]. In addition, yunnaneic acid D was identified according to its molecular formula (C_27_H_24_O_12_), ion (*m*/*z* 539.1201) and its fragment ions at *m*/*z* 169.0291/197.0237/311.0569 [23,24].

Finally, 4′-*O*-methylgallocatechin-(4->8)-4′-*O*-methylepigallocatechin were also detected. These compounds showed a dimeric ion at *m*/*z* 637.1575, and fragment ions at *m*/*z* 331.0625/375.0513/577.1390 [25].

### 2.2. Hypoxia Induced PC12 Cell Injury

**Figure 2:**
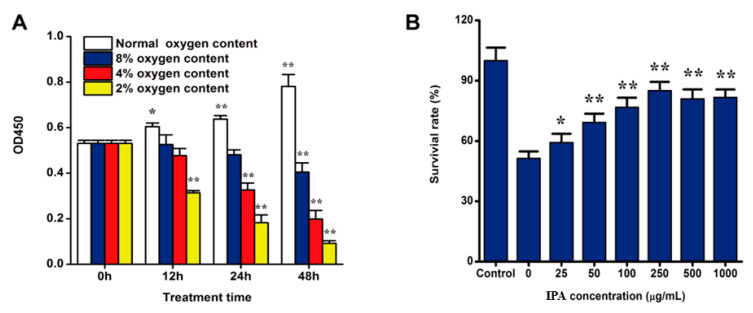
Hypoxia induced PC12 cell injury and IPA promote the survival of hypoxia-damaged PC12 cells. (**A**) Effect of oxygen contents and treatment time on the survival of PC12 cells; (**B**) Protective effect of IPA on hypoxia-damaged PC12 cells, as determined by CCK-8 assay. * *p* < 0.05, ** *p* < 0.01 indicates a significant difference versus the control group.

At first, we built up a hypoxia-induced damage model of PC12 cell (Figure 2A), and results showed hypoxia could induce PC12 cells injury and it was time-dependent and oxygen content-dependent. Hypoxia under 4% oxygen content for 24 h could result in significant hypoxia injury for PC12 cells and the values of OD_450_ could decrease about 50%. Further validation experiments indicated such condition was stable and repeatable.

### 2.3. IPA Promotes the Survival of Hypoxia-Damaged PC12 Cells

We investigate if IPA suppresses PC12 cells growth in normoxia. After PC12 cells were treated with different concentrations of IPA (25–2000 µg/mL) for 48 h, the CCK-8 assay showed that IPA did not inhibit PC12 cells growth within the treatment concentration (data shown in Figure S2). Then we investigated the protective effects of IPA isolated from ABSC on hypoxia-induced PC 12 cells. As shown in Figure 2B, the CCK-8 assay showed that IPA could promote the survival of PC12 cell under hypoxia oxygen conditions significantly (*p* < 0.01). When hypoxia-damaged PC12 cells were incubated with 25–250 μg/mL IPA, the viability of PC12 cell was remarkably (*p* < 0.05) increased with a dose-dependent manner. When the PC12 cells were protected with IPA at the concentration of 250 μg/mL for 24 h, the cell viability increased to the highest point, up to 85.08 ± 4.34% (*p* < 0.01).

### 2.4. IPA Decreases the Number of Apoptotic and Necrotic Hypoxia-Damaged Cells

According to above results, the viabilities of PC12 cells significantly increased after 24 h hypoxia treatment with oxygen contents at 4% with the concentration of 250 μg/mL. Therefore, we chose to use 250 μg/mL IPA for the optional concentration for additional studies. Since high concentration of IPA (over 250 µg/mL) tended to weaken the protective effects of IPA while low concentration of IPA (100 μg/mL) showing increasing protective effects on hypoxia-damaged PC12 cells, so 100 μg/mL of IPA was also selected for the low dose group.

**Figure 3:**
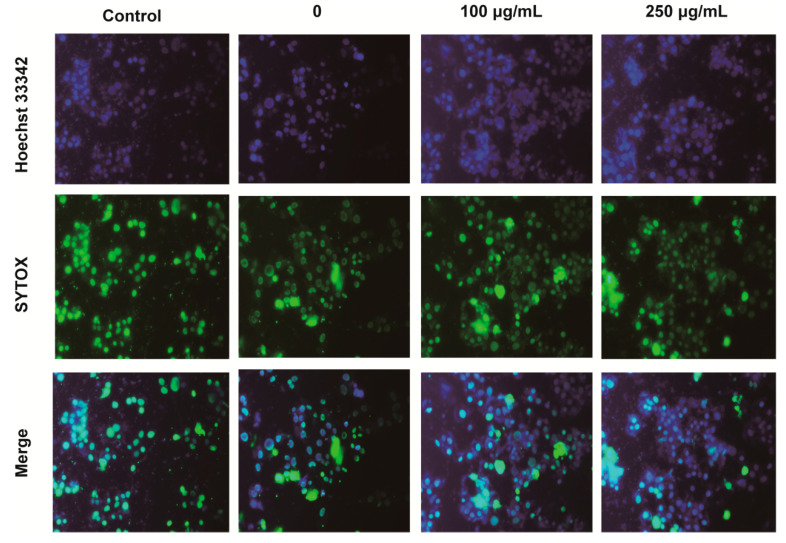
Fluorescence micrographs of PC12 cells stained with Hoechst 33342 and SYTOX green staining (original magnification, ×100).

In the Hoechst33342 and SYTOX green staining test, apoptotic PC12 cells were marked by strong blue fluorescence, normal cells displayed weak blue fluorescence, and necrotic cells presented strong green fluorescence. As shown in Figure 3, compared with the control cells, hypoxia induced the increasing numbers of necrotic cells (green fluorescence), we also observed an increase in the number of viable cells and other apoptotic features, such as fragmented chromatin under hypoxia. However, IPA treatment could decrease the number of apoptotic and necrotic PC12 cells with the stronger blue fluorescence and weaken green fluorescence. The findings indicated that IPA showed protective effects on hypoxia-damaged PC12 cells, as the decreased cellular apoptosis characteristics showing chromatin condensation, cellular shrinkage, apoptotic bodies, and cytoplasmic condensation.

### 2.5. IPA Protects Hypoxia-Damaged PC12 Cells from Overproduced ROS Damage and Mitochondrial Damage

**Figure 4:**
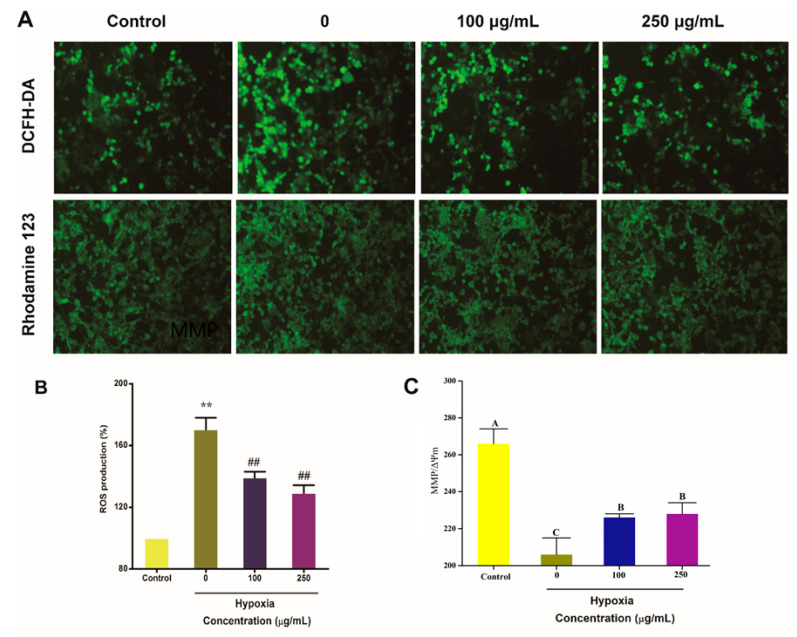
IPA protects hypoxia-damaged PC12 cells from overproduced ROS and mitochondrial damage. (**A**) Representative images showing the effects of IPA on reducing the overproduced ROS by DCFH-DA staining and the mitochondrial membrane potential (ΔΨm) in hypoxia-damaged PC12 cells as determined by Rhodamine-123 (original magnification, × 100). (**B**) The ROS production for each PC12 cell treatment. (**C**) The ΔΨm for each PC12 cell treatment. Data are expressed as the mean ± SD (n = 3; one-way analysis of variance followed by the *T*-test). The different uppercase letters A, B, C indicate significant differences, all experiments were conducted in triplicate. ** *p* < 0.01 indicates a significant difference versus the control group. ## *p* < 0.01 indicates a significant difference versus the 0 group under hypoxia condition.

Previous studies have shown that hypoxia can result in increased ROS production in cell. To examine whether IPA protect PC12 cells from hypoxia-damaged injuries, PC12 cells were treated with 100 μg/mL and 250 μg/mL IPA for 24 h under 4% oxygen content, respectively, and intracellular ROS levels were examined in PC12 cells (Figure 4A). Compared with normal PC12 cells (control group), ROS was dramatically enhanced to 170.34 ± 7.64% (*p* < 0.01) in hypoxia-treated PC12 (Figure 4B). However, IPA treatment could greatly downregulate the increased ROS formation induced by hypoxia (*p* < 0.01; Figure 4B). The ROS production decreased to 139.15 ± 3.92% and 129.17 ± 5.05% when hypoxia-damaged PC12 cells were protected with 100 μg/mL and 250 μg/mL IPA for 24 h, respectively.

We also examined mitochondrial membrane potential (ΔΨm) in PC12 cells from different groups. Compared with normoxic PC12 cells (the control group), ΔΨm levels were greatly reduced 22.44 ± 3.15% in the hypoxia-treated PC12 cells (*p* < 0.01; Figure 4C). However, IPA treatment could significantly reverse this effect (*p* < 0.01; Figure 4C). When hypoxia-damaged PC12 cells were protected with 100 μg/mL and 250 μg/mL IPA for 24 h, the ΔΨm decreased to 15.00 ± 0.56% and 14.45 ± 1.95%, respectively, than that of the control group.

### 2.6. IPA Reduces Hypoxia-Induced Apoptosis Rate of PC12 Cells

**Figure 5:**
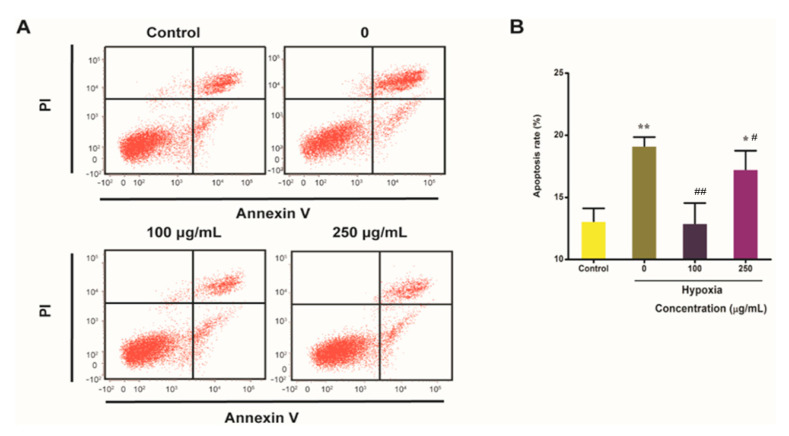
IPA reduce hypoxia-induced apoptosis rate of PC12 cells. (**A**) Cell apoptosis rate was determined by flow cytometry. (**B**) The apoptosis rate for each PC12 cell treatment. Data are expressed as the mean ± SD (n = 3; one-way analysis of variance followed by the *T*-test). All experiments were conducted in triplicate. * *p* < 0.05, ** *p* < 0.01 indicates a significant difference versus the control group. # *p* < 0.05, ## *p* < 0.01 indicates a significant difference versus the 0 group under hypoxia condition.

Owing to mitochondrial dysfunction can lead to apoptosis, we examined whether IPA protected hypoxia-induced apoptosis in PC12 cells. PC12 cells were cultured with 100 μg/mL and 250 μg/mL IPA for 24 h. Then apoptosis was measured in the sorted PC12 cells by flow cytometry. Compared with the control PC12 cells, apoptosis of PC12 cells was significantly enhanced to 19.14 ± 0.71% under hypoxia challenge with 4% oxygen content (*p* < 0.01; Figure 5B). However, IPA could reduce this increased apoptosis caused by hypoxia (*p* < 0.01; Figure 5B). When compared with the PC12 cells without IPA protection under hypoxia, apoptosis was significantly reduced 1.48 times in the hypoxia-treated PC12 cells with 100 μg/mL IPA protection (*p* < 0.01; Figure 5B).

As well, we confirmed IPA’s effects on cell apoptosis using Annexin-FITC and propidium iodide (PI) staining (data shown in Figure S3). Non-apoptotic cells were marked by green nuclear fluorescence when stained with Annexin V-FITC, and apoptotic PC12 cells were marked by strong red fluorescence when stained with PI (*p* < 0.01; Figure S3). As shown in Figure S3, there was an increase in the number of annexin V-FITC-stained cells and decrease in the number of PI-stained cells following IPA treatment compared to the group without IPA treatment under hypoxia conditions. The results confirmed that IPA treatment could protect hypoxia-damaged PC12 cells from apoptosis.

## 3. Discussion

Exposure to hypoxia showed the increased oxidative stress and could induce the overproduction of ROS [26]. ROS is considered to be a major macrophage effector mechanism in cells. Under hypoxia mitochondria are severely affected by decreased oxygen availability and thus different adaptive changes occur in the organelle morphology and metabolism, resulting in modified composition/assembly of subunits of the respiratory chain, oxidative phosphorylation, and reductive carboxylation [27,28]. These processes allow ROS increment at low oxygen levels. ROS release through oxidative burst is an inducing apoptosis for hypoxia-injured cells. Our findings indicate that hypoxia induces ROS over-produced in hypoxia-damaged PC12 cells that may induce cells apoptosis under hypoxia, but IPA treatment could significantly decrease the levels of ROS and presented the anti-hypoxia activity on PC12 cells. Phenolic antioxidants function as terminators of free radicals and chelators of metal ions that are capable of catalyzing lipid peroxidation [29], and the anti-hypoxia protective effects of IPA could due to its antioxidant capacities and radical scavenging properties by rapidly donating a hydrogen atom to radicals, since phenolic antioxidants such as ECG derivative, yunnaneic acid D, and 4′-*O*-methylgallocatechin-(4->8)-4′-*O*-methylepigallocatechin have been discovered in IPA extractions (75% ethanol extract) by HPLC-Q-TOF-MS in ABSC. Such results are in corroboration with that of Dhar et al. [30], who found that there is an elevated level of free radicals and lipid peroxidation in seven-day exposure to hypobaric hypoxia-induced rat. They also found that administration of antioxidant-rich formulated materials, consisting of the extracts from the roots of *Hippophae rhamnoides*, *Prunus armeniaca*, and *Rhodiola imbricate*, could reduce the free radical level and lipid peroxidation during exposure. Concerning its relative bigger peak area and important bioactivities of these phenolic antioxidants, more work is required to elucidate the molecular and clinical preventive effects of IPA in ABSC on promoting human health.

Increasing studies have shown that hypoxia induces cell apoptosis, and the structure and function abnormalities of mitochondria are closely related to cell apoptosis [31]. Dispersion of the mitochondrial membrane potential plays an extremely important role in process of cell death or apoptosis [32]. It was reported that mitochondrial dysfunction in neurons is the major cause of hypoxia-induced brain damage, which was caused by the reduced mitochondrial transmembrane potential and elevated ROS generation [33,34]. Here, we found that hypoxia enhanced ROS generation and reduced the ΔΨm in PC12 cells, indicating that hypoxia induced mitochondrial damage. However, treatment with IPA obviously alleviate the mitochondrial damage in hypoxia-induced PC12 cells and such protection could due to its ability to scavenge the ROS, indicating that the anti-hypoxia effects of IPA are closely due to its antioxidant activity [35]. It was reported that the damage to mitochondrial membrane integrity induced by hypoxia has been shown to activate autophagy, resulting in apoptosis of neurons [36,37,38]. Whether the protective effects of IPA on hypoxia-injured PC12 cells is involved in autophagy process requires further investigation.

Exposure to hypoxic conditions for humans is very common in life. Some factors such as strenuous exercise, high-altitude environment, some diseases, and so on, could threaten people suffering hypoxia. Especially, hypoxia in high altitude continuously affects physical and mental performances and it is known to cause cognitive and mental dysfunctions such as memory deficits, motor impairment, and hypophagia [39,40]. Diverse arrays of physiological and psychological responses are instigated by the unique physical and environmental factors of cold arid high-altitude regions of the world [41]. The plateau is characterized by chillness and oxygen shortage. For organisms inhabiting this area, hypoxia and coldness exert strong selective pressures that drive evolution of morphological and physiological characteristics to adapt to these stresses [42]. ABSC is a native macro fungus in Qaidam Basin (Qinghai, China) and it has been consumed as an edible and medicinal fungus for supplements in locals. To ameliorate these high-altitude health ailments including cognitive impairment and loss of memory, it is very significant to develop novel prophylactic foods or drugs with anti-hypoxia activity using the indigenous botanical resources of this region. Chemical drugs with anti-hypoxic effects—such as amphetamine, acetazolamide, dexamethasone, and nimodipine—have not been widely used because of their side effects. Therefore, it is essential and urgent for researchers to improve the understanding of hypoxia adaptation of organisms and find natural, efficiency, nontoxic, easy-available bioactive compounds, medicinal food, and medicines to not only help people adapt to the high-altitude environment, prevent and cure hypoxic injure, but also make contribution to polar medical science, sports science and plateau military science. Previously, we mainly focus on investigating the anti-hypoxia activities and its mechanism of intracellular polysaccharides and extracellular polysaccharides isolated from ABSC, and such polysaccharides were proven to show anti-hypoxia activities at experiment animals and cell levels [18,19]. In this work, the IPA extracted from ABSC also proved to show anti-hypoxia activity. IPA, at the concentration of 100 μg/mL and 250 μg/mL could significantly reduce hypoxia-induced damage in PC12 cells compared with the control group (*p* < 0.01). Further study indicated IPA could protect PC12 cells from hypoxia-induced damage by decreasing the overproduced intracellular ROS, improving ΔΨm and reducing apoptosis rate. These researches of exploring the bioactivities of potential components derived from ABSC informed us that its fermentation products could be a potential, natural, and efficient resources to help people adapt to the high-altitude environment, as well as prevent and cure hypoxic injury. The fermentation process could make it possible to produce larger amounts of biomass and fermentation products with potential anti-hypoxia activities. However, more attention could be paid to study anti-hypoxia activity and its molecular mechanisms.

## 4. Materials and Methods

### 4.1. Materials

The fruiting bodies of ABSC macrofungus collected from Qinghai Province, China, were provided by the Wild Plants Resources Institute of Qinghai Academy of Agriculture and Forestry Science (Xining, China) and identified by Prof. Y.C. Jiao of Qinghai University. The inclining strain (ZJU-CDMA-12) of ABSC was cultured to prepare liquid seed culture broth following the processes as described before [18]. The secondary seed medium was composed of 20 g/L fructose, 20 g/L maltose, 5 g/L peptone, 1 g/L vitamin B1, 0.6 g/L MgSO_4_, and 0.14 g/L CaCl_2_. The fermentation broth for polyphenols accumulation was consisted of 10.00 g/L glucose, 18.00 g/L fructose, 10.00 g/L sucrose, 18.00 g/L maltose, 5.00 g/L peptone, 0.10 g/L CaCl_2_, 0.03 g/L vitamin C, 1.00 g/L KH_2_PO_4_·H_2_O, 0.50 g/L MgSO_4_·7H_2_O, pH 2.95. The fermentation process for polyphenols accumulation was conducted in a shaking incubator operated at 130 ± 5 rpm at 20 ± 2 °C for 12 d in the dark. After the fermentation process, the broths were filtrated with dried filter paper. The deposited mycelia were washed with distilled water three times and they were vacuum freeze-drying for 36 h. The extraction method of IPA was modified with reference to Gogoi [43]. After being grinded in aseptic condition, the dried mycelia were extracted with 50 mL 75% (*v/v*) ethanol by ultrasound-assisted extraction at 45 °C for 1 h and repeated the extraction for 3 times, then separated by centrifugation at 13,000 rpm and concentrated at 45 °C under the reduced pressure (0.02 kPa) to get the IPA extractions.

### 4.2. Determination of IPA Content

IPA content was determined by using Folin–Ciocalteu reagent (Macklin, Shanghai, China) [44]. Briefly, 0.5 mL of diluted sample was mixed with 2 mL of 2 N Folin–Ciocalteu phenol reagent and 2.5 mL of 7.5% (*w/v*) sodium carbonate solution. Reaction mixtures were incubated at room temperature for 30 min in darkness. The absorbance was measured at 765 nm. A gallic acid standard curve with a linear range (0–100 μg/mL) was prepared and results were expressed as mg of gallic acid equivalents (GAE)/g of soluble fraction.

### 4.3. Isolation and Characterization of IPA

#### 4.3.1. Isolation Procedure

The fermentation broth (5 L) was concentrated under reduced pressure to a volume of 0.5 L and then extracted with 2.5 L 75% (*v/v*) ethanol as described in 4.1. The extracts were combined, centrifuged at 1500 g for 15 min and the supernatant was evaporated at 45 °C under 0.02 MPa and then filtered with a 0.2 μm polytetrafluoroethylene (PTFE) filter to obtain the IPA extracts. The IPA extracts were freeze-dried for further high performance liquid chromatography (HPLC) quadrupole time-of-flight mass spectrometry (HPLC-Q-TOF-MS) analysis and cell culture studies. Prior to HPLC-Q-TOF-MS analysis, the freeze-dried mixture was rehydrated with 1 mL of Milli-Q water which was boiled for 3 min and then cooled to room temperature to reduce the dissolved air, especially CO_2_.

#### 4.3.2. HPLC-Q-TOF-MS Analysis

The identification of IPA was performed in electrospray ionization (ESI-) mode on an Agilent 6500 HPLC-Q-TOF-MS apparatus connected to an Agilent 1200 series HPLC system (Agilent Technologies, Santa Clara, CA, USA). IPA solutions were separated on a Zorbax SB-C18 column (150 mm × 460 mm, 5-Micron, Agilent Technologies, Santa Clara, CA, USA) at 30 °C. A gradient with a flow rate of 0.3 mL/min was used, with solvent A (formic acid 0.1% in Milli-Q water) and solvent B (formic acid 0.1% in methanol) under the following conditions: starting with 95-5% A-B, ramping to 5-95% A-B in 15 min, held at 5-95% A-B for 15.5 min, return to the initial conditions 95-5% A-B at 16 min and reconditioning the system at 95-5% A-B for 25 min. The absorbance of the efflux was monitored at 220 nm by DAD (diode array detector). The Q-TOF-MS was operated in full-scan mode from 100 to 1000 for monitoring negative ions. Nitrogen was used as the nebulizing and drying gas. Operating conditions were as follows: gas temperature, 350 °C; gas flow, 10 L/min; fragmentor, 110 V; capillary voltage, 4000 V; skimmer, 65 V; OCT 1 RF Vpp, 750 V. The sample collision energy was set at 10, 20, 30, and 40 V. Mass spectra obtained were qualitatively analyzed by Agilent LCMS-QTOF Mass Hunter Acquisition Software version B 06.00 (Agilent Technologies, Santa Clara, CA, USA). All references were obtained by searching databases, such as PubMed of the US National Library Medicine, Reaxy databases, SciFinder Scholar of American Chemical Society and the Chinese National Knowledge Infrastructure (CNKI) of Zhejiang University, China.

### 4.4. Anti-Hypoxia Activity

#### 4.4.1. Cell Culture and Cytotoxicity Assay

PC12 cells (purchased from Cell Bank of the Chinese Academy of Sciences, Shanghai, China) were cultured in Dulbecco’s modified Eagle medium (DMEM medium, Corning, NY, USA) supplemented with 10% fetal bovine serum (FBS, GIBCO/Invitrogen, Carlsbad, USA) and 2 mM L-glutamine and 1% penicillin-streptomycin solution (GIBCO/Life Technologies, Carlsbad, USA) at 37 °C and 5% CO_2_ atmosphere. The culture medium was changed every 2 days. Before a confluent monolayer appeared, subculturing cell process was carried out. After 24 h incubation in a 96-well plate (1 × 10^4^ cells/well), the PC12 cells were cultured in the presence of different concentrations of IPA for 48 h. After incubation, then the cell viability was tested by Cell Counting Kit-8 (CCK-8) (Beyotime, Shanghai, China) assays to evaluate IPA cytotoxicity in PC12 cells following the instructions provided by the CCK-8 kit. As for the CCK-8 assay, briefly, the cells added with CCK-8 were incubated for 2 h at 37 °C in the dark, then the absorbance at 450 nm was measured with a spectrophotometer. The cell viability was calculated by the equation

Cell viability (%) = A_sample_/A_control_ × 100%

where A_control_ = absorbance of control group (without IPA addition) minus blank well and A_sample_ = absorbance of IPA-treated group minus blank well.

#### 4.4.2. Treatment Condition

Hypoxia experiments were performed in a hypoxia box (95% N_2_ + 5% CO_2_ were charged into the box at 1 L/min flow continuously, oxygen concentration < 0.1%) at 37 °C. PC12 cells were incubated in hypoxia or normoxia chambers for indicated time points (0, 12, 24, and 48 h, respectively) in serum free medium containing 1% penicillin-streptomycin. The cell viabilities under hypoxia condition at different culture times were determined by CCK-8.

#### 4.4.3. CCK-8 Assay

For researching the protective effects of IPA on the hypoxia-damaged PC12 cells, PC12 cells at the density of 5 × 10^3^ cells/mL were plated in one well of 96-well plate and cultivated for 24 h under normoxia chambers as determined in Section 4.4.1, then the supernatant was removed and 100 µL of culture mediums containing different concentration of IPA were added into the protection groups under hypoxia-damaged conditions for incubation. After then, the cell viability was tested by CCK-8 assays.

#### 4.4.4. Hoechst 33342 and SYTOX Staining

Based on the results of the CCK-8 assay, PC12 cells were divided into four groups: control (under normoxia), 0 (under hypoxia), IPA (100 μg/mL, under hypoxia) and IPA (250 μg/mL, under hypoxia), cells were treated as described before. Hoechst 33342 staining (Beyotime, Shanghai, China) and SYTOX green staining (Invitrogen, Thermo Fisher, Waltham, MA, USA) were used to investigate the protection of IPA on hypoxia-induced PC12 cells. In brief, at the end of incubation as described in Section 4.4.2, 5 μg/mL Hoechst 33342 staining solutions and 5 μM SYTOX green staining were added and incubated with PC12 cells for 30.0 min at 37 °C in dark, followed by washing with PBS for several times. Then, the cells were observed under fluorescence microscope (Olympus, Tokyo, Japan) with double excitation wavelengths at 346 nm (blue) and 525 nm (green), respectively. Images were processed and fluorescence intensity was calculated using Image J software v 1.8.2.

#### 4.4.5. Intracellular ROS Measurement and Mitochondrial Membrane Potential (∆ψm)

Intracellular ROS was estimated by using a fluorescent probe, 2′,7′-dichlorodihydrofluorescein diacetate (DCFH-DA) (Beyotime, Shanghai, China) [45]. In brief, at the end of incubation as described in Section 4.4.2, the culture medium was removed and washed with PBS. Finally, DCFH-DA (final concentration 10 μM/L) was added in the dark and incubated at 37 °C for 30 min. Then the medium was removed and washed twice with PBS. Mitochondrial membrane potential (∆ψm) in PC12 cells was determined using the fluorescence probe Rhodamine-123 (Beyotime, Shanghai, China) for the depolarization of ∆Ψm. After treatment, the cells were incubated with 5 μM Rhodamine-123 for 30 min at 37 °C in darkness. Before observation, they were washed for three times with PBS. The stained cells were observed under a fluorescence microscopy (Olympus, Tokyo, Japan), the fluorescence was determined by flow cytometry (Becton Dickinson, Franklin Lakes, NJ, USA), and the results were presented as a percentage of control groups.

#### 4.4.6. Detection of Cell Apoptosis

The apoptosis rate was evaluated using the Annexin V-FITC/PI Apoptosis Detection kit according to the instructions from the manufacturer (Yesen, Shanghai, China). Briefly, at the end of incubation as described in Section 4.4.2, PC12 cells (1 × 10^6^) were collected and centrifuged for 5 min at 300 g, the deposit were washed with PBS, and incubation with the annexin-V FITC (5 µL) and PI staining solution (10 µL) in binding buffer at room temperature for 10 min in the dark, then tested by flow cytometry (Becton Dickinson, Franklin Lakes, NJ, USA) within 1 h and data were analyzed by the Flow Jo 10 software v 10.2 After tested, the stained cells were immediately observed under a fluorescence microscopy (Olympus, Tokyo, Japan) with double excitation wavelengths at 525 nm (green) and 535 nm (red), respectively.

### 4.5. Statistical Analysis

All data were expressed as the mean ± SD. All determinations were based on three replicate samples in vitro experiment. Fisher’s least significance difference (LSD) test was performed using IBM SPSS Statistic 9 (IBM Corp., Armonk, NY, USA). The Statistica software (trial version 6.0, StatSoft, Tulsa, OK, USA) was employed for regression and graphical analyses of data obtained.

## 5. Conclusions

In this study, IPA were extracted from ABSC and the structure characterization and the anti-hypoxia activity of IPA on PC12 cells were researched. The results of HPLC-Q-TOF-MS illustrated that five kinds of IPA were isolated from ABSC, including (−)-epicatechin gallate, arabelline, yunnaneic acid D, 2′-*O*-*p*-hydroxybenzoyl-6′-*O*-*trans*-caffeoylgardoside, and 4′-*O*-methylgallocatechin-(4->8)-4′-*O*-methylepigallocatechin. The results of anti-hypoxia activity indicated that IPA extracted from ABSC proved to show anti-hypoxia activity on hypoxia-damaged PC12 cells. Hypoxia enhanced ROS generation and reduced the ΔΨm in PC12 cells, resulting in the inhibition of survival and induction of apoptosis in PC12 cells. However, compared with the control group, IPA with the concentration of 100 μg/mL and 250 μg/mL could significantly reduce hypoxia-induced damage in PC12 cells by decreasing the overproduced intracellular ROS, improving ΔΨm and reducing apoptosis rate. The findings indicated that the IPA from ABSC could be a novel health-promoting product with anti-hypoxia activities. However, the structure information and the mechanism of its anti-hypoxia effect are still unconfirmed, further studies should be focused on this.

## Figures and Tables

**Table 1:** HPLC-Q-TOF-MS characterization of main compounds in IPA from ABSC

		[M-H]^−^(*m/z*)					
Peak no.	Rt (min)	Detected	Expected	Error (ppm)	Formula	Identification	Fragments (*m/z*)
1	6.629	673.1795	674.612	2.3	C_32_H_34_O_16_	arabelline	321.0422/381.0646/441.0862
2	6.637	441.0827	442.379	−1.9	C_22_H_18_O_10_	(−)-epicatechin gallate	251.0347/295.0243/381.0627
3	8.827	657.1778	656.597	1.8	C_32_H_32_O_15_	2′-*O*-p-hydroxybenzoyl-6′-*O*-trans-caffeoylgardoside	455.0980/561.1363/579.1468
4	9.659	539.1201	540.480	0.4	C_27_H_24_O_12_	yunnaneic acid D	169.0291/197.0237/311.0569
5	10.012	637.1575	638.582	2.4	C_32_H_30_O_14_	4′-*O*-methylgallocatechin-(4->8)-4′-*O*-methylepigallocatechin	331.0625/375.0513/577.1390

## Supplementary Materials

The following are available online, Figure S1. the characteristic ion mass spectrometries of arabelline (A), (−)-epicatechin gallate(B), 2′-O-p-hydroxybenzoyl-6′-O-trans-caffeoylgardoside (C), yunnaneic acid D (D), 4′-O-methylgallocatechin-(4->8)-4′-O-methylepigallocatechin (E). Figure S2. The cytotoxicity of IPA on PC12 cells under normoxia. Figure S3. Representative images showing the protective effects of IPA on cell apoptosis using Annexin-FITC and Propidium Iodide (PI) staining. Table S1. Data information of base peak chromatogram of IPA.
