# Supplementary material for: Phenolic Acids-Rich Fractions from Agaricus bitorguis (Quél.) Sacc. Chaidam ZJU-CDMA-12 Mycelia Modulate Hypoxic Stress on Hypoxia-Damaged PC12 Cells
Source: Molecules. 2020 Oct 21;25(20):4845. doi: 10.3390/molecules25204845 (PMC7587959; doi:10.3390/molecules25204845)
Supplement: Supplementary file 1 [file molecules-25-04845-s001.zip › supplementary files/Fig. S1. the characteristic ion mass spectrometries of arabelline (A), (-)-epicatechin gallate(B), 2'-O-p-hydroxybenzoyl-6'-O-trans-caffeoylgardoside (C), yunnaneic acid D (D), 4'-O-methylga.pdf]

A

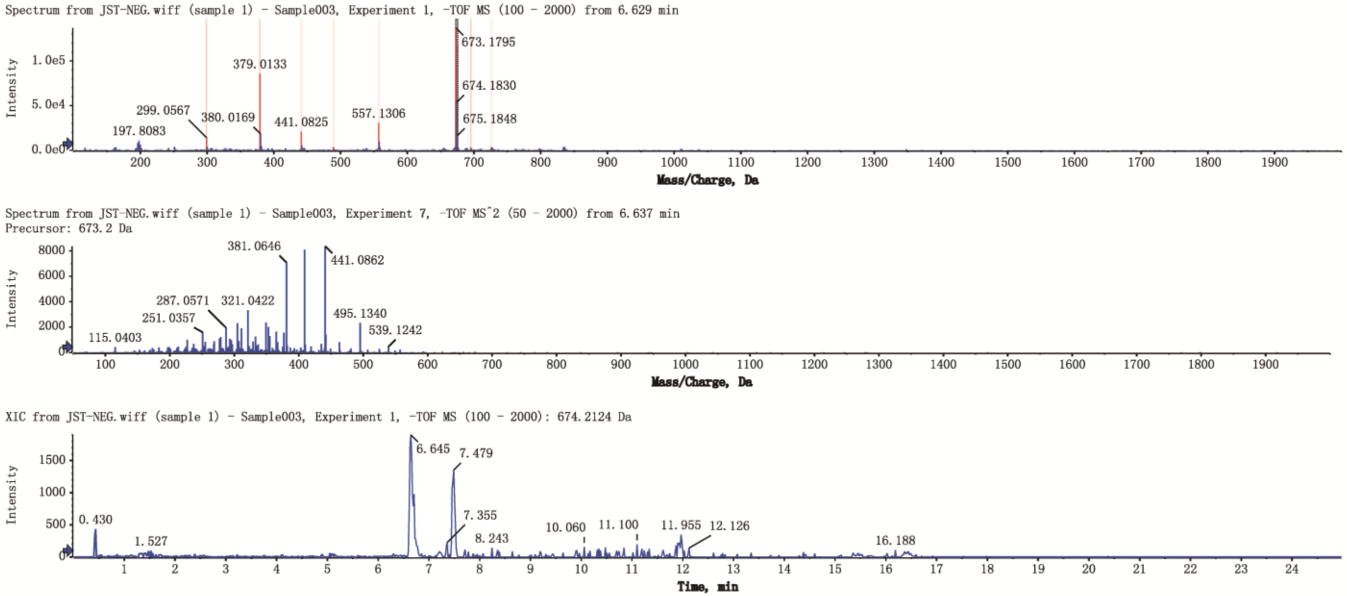
Spectrum from JST-NEG.wiff (sample 1) - Sample003, Experiment 1, -TOF MS (100 - 2000) from 6.629 min
Intensity
1.0e5
5.0e4
0.0e0
673.1795
379.0133
674.1830
299.0567
380.0169
441.0825
557.1306
675.1848
197.8083
Mass/Charge, Da
Spectrum from JST-NEG.wiff (sample 1) - Sample003, Experiment 7, -TOF MS^2 (50 - 2000) from 6.637 min
Precursor: 673.2 Da
8000
6000
4000
2000
381.0646
441.0862
287.0571
321.0422
251.0357
495.1340
539.1242
115.0403
Mass/Charge, Da
XIC from JST-NEG.wiff (sample 1) - Sample003, Experiment 1, -TOF MS (100 - 2000): 674.2124 Da
1500
1000
500
6.645
7.479
7.355
0.430
1.527
8.243
10.060
11.100
11.955
12.126
16.188
Time, min

B

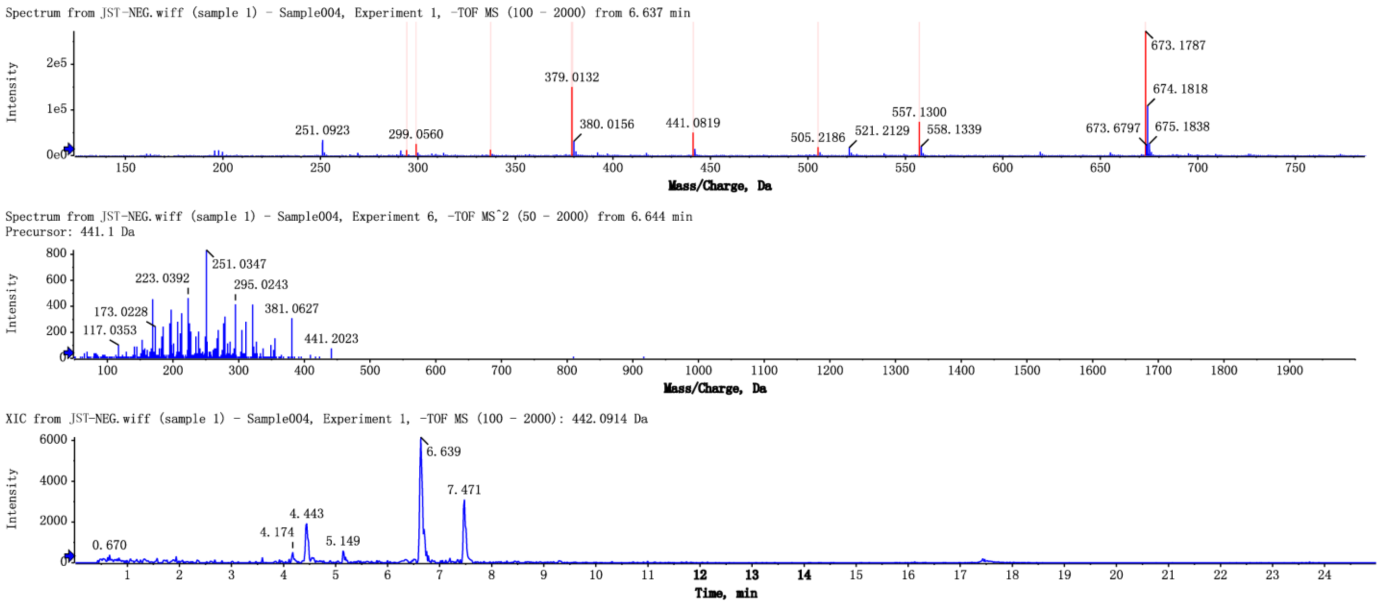

Spectrum from JST-NEG.wiff (sample 1) - Sample004, Experiment 1, -TOF MS (100 - 2000) from 6.637 min
Intensity
Mass/Charge, Da
379.0132
380.0156
441.0819
251.0923
299.0560
505.2186
521.2129
557.1300
558.1339
673.1787
674.1818
673.6797
675.1838
Spectrum from JST-NEG.wiff (sample 1) - Sample004, Experiment 6, -TOF MS^2 (50 - 2000) from 6.644 min
Precursor: 441.1 Da
251.0347
223.0392
295.0243
173.0228
117.0353
381.0627
441.2023
XIC from JST-NEG.wiff (sample 1) - Sample004, Experiment 1, -TOF MS (100 - 2000): 442.0914 Da
Time, min
6.639
7.471
4.443
4.174
5.149
0.670

C

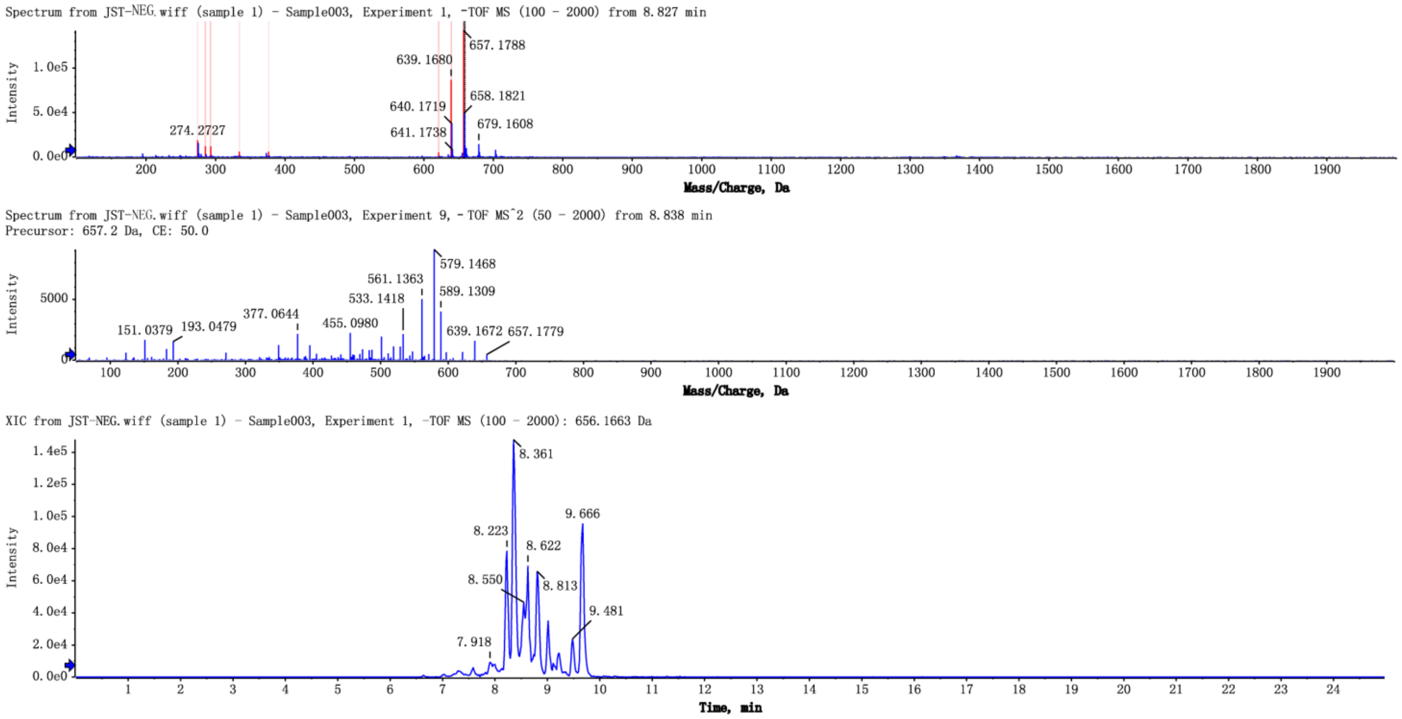
Spectrum from JST-NEG.wiff (sample 1) - Sample003, Experiment 1, -TOF MS (100 - 2000) from 8.827 min
Intensity
657.1788
639.1680
658.1821
640.1719
679.1608
641.1738
274.2727
Mass/Charge, Da
Spectrum from JST-NEG.wiff (sample 1) - Sample003, Experiment 9, - TOF MS^2 (50 - 2000) from 8.838 min
Precursor: 657.2 Da, CE: 50.0
579.1468
561.1363
589.1309
533.1418
377.0644
455.0980
151.0379
193.0479
639.1672
657.1779
Mass/Charge, Da
XIC from JST-NEG.wiff (sample 1) - Sample003, Experiment 1, -TOF MS (100 - 2000): 656.1663 Da
8.361
9.666
8.223
8.622
8.550
8.813
9.481
7.918
Time, min

D

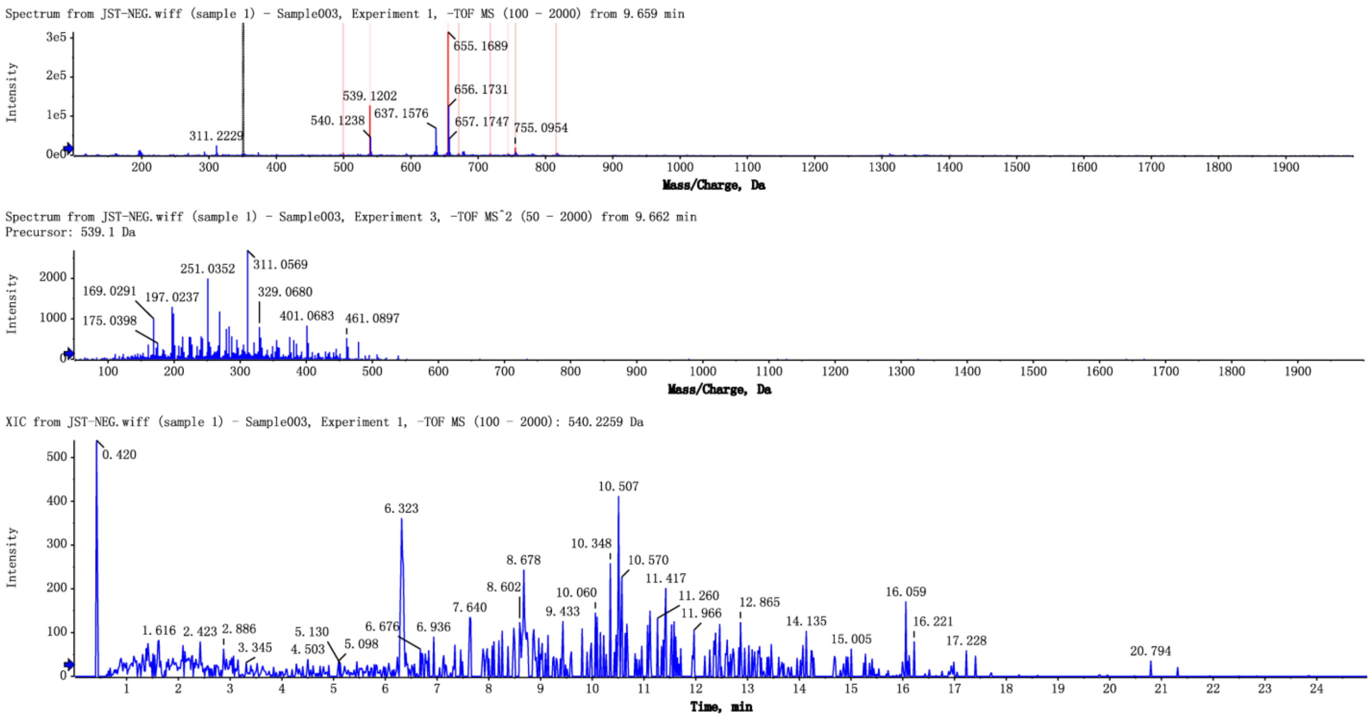

Spectrum from JST-NEG.wiff (sample 1) - Sample003, Experiment 1, -TOF MS (100 - 2000) from 9.659 min
Intensity
Mass/Charge, Da
655.1689
539.1202
656.1731
637.1576
540.1238
657.1747
755.0954
311.2229
Spectrum from JST-NEG.wiff (sample 1) - Sample003, Experiment 3, -TOF MS^2 (50 - 2000) from 9.662 min
Precursor: 539.1 Da
Intensity
Mass/Charge, Da
311.0569
251.0352
329.0680
169.0291
197.0237
401.0683
461.0897
175.0398
XIC from JST-NEG.wiff (sample 1) - Sample003, Experiment 1, -TOF MS (100 - 2000): 540.2259 Da
Intensity
Time, min
0.420
10.507
6.323
10.348
10.570
8.678
11.417
8.602
11.260
10.060
11.966
9.433
12.865
7.640
16.059
14.135
16.221
1.616
2.423
2.886
6.676
6.936
15.005
17.228
5.130
3.345
5.098
4.503
20.794

E

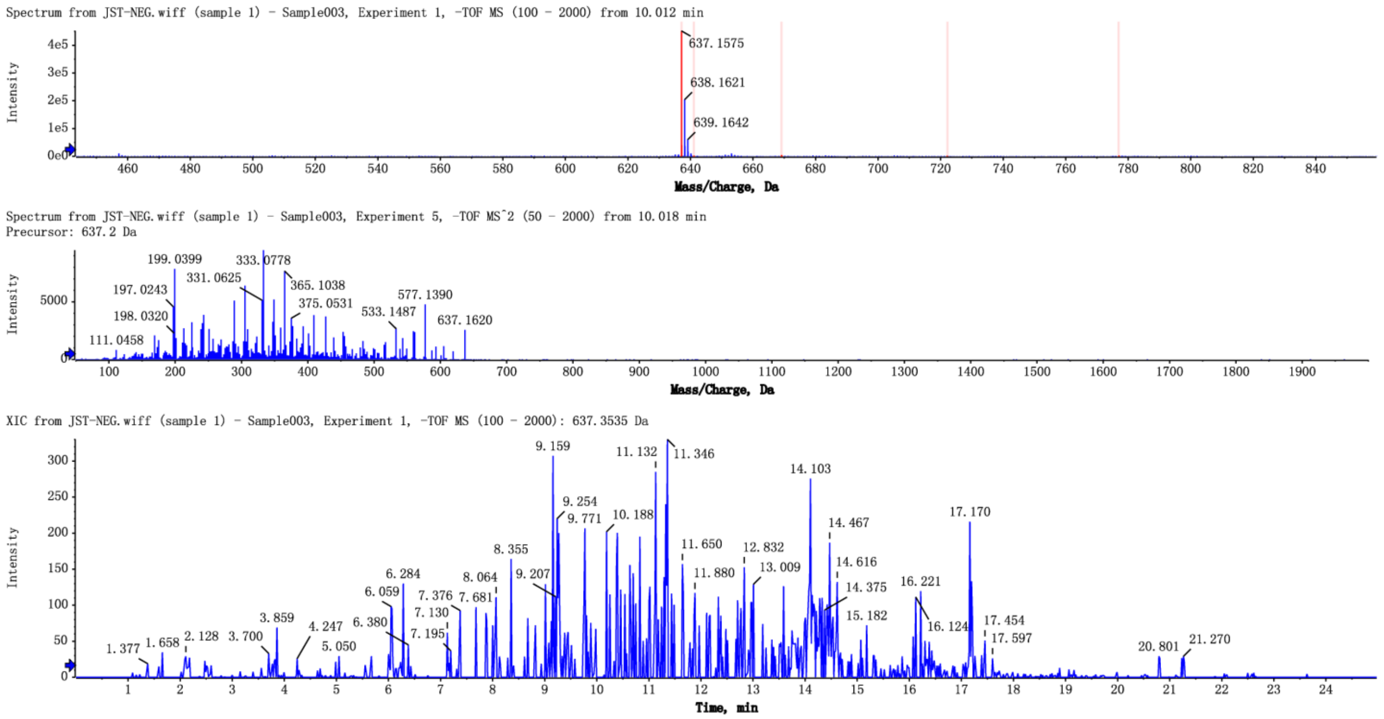
Spectrum from JST-NEG.wiff (sample 1) - Sample003, Experiment 1, -TOF MS (100 - 2000) from 10.012 min
637.1575
638.1621
639.1642
Intensity
Mass/Charge, Da
Spectrum from JST-NEG.wiff (sample 1) - Sample003, Experiment 5, -TOF MS^2 (50 - 2000) from 10.018 min
Precursor: 637.2 Da
199.0399
333.0778
331.0625
197.0243
365.1038
577.1390
375.0531
198.0320
533.1487
637.1620
111.0458
Intensity
Mass/Charge, Da
XIC from JST-NEG.wiff (sample 1) - Sample003, Experiment 1, -TOF MS (100 - 2000): 637.3535 Da
Intensity
Time, min

Figure S1. the characteristic ion mass spectrometries of arabelline (A), (-)-epicatechin gallate(B), 2'-O-p-hydroxybenzoyl-6'-O-trans-caffeoylgardoside (C), yunnaneic acid D (D), 4'-O-methylgallocatechin-(4->8)-4'-O-methylepigallocatechin (E)
