# Supplementary material for: Phenolic Acids-Rich Fractions from Agaricus bitorguis (Quél.) Sacc. Chaidam ZJU-CDMA-12 Mycelia Modulate Hypoxic Stress on Hypoxia-Damaged PC12 Cells
Source: Molecules. 2020 Oct 21;25(20):4845. doi: 10.3390/molecules25204845 (PMC7587959; doi:10.3390/molecules25204845)
Supplement: Supplementary file 1 [file molecules-25-04845-s001.zip › supplementary files/Fig. S2 the cytotoxicity of IPA on PC12 cells under normoxia.pdf]

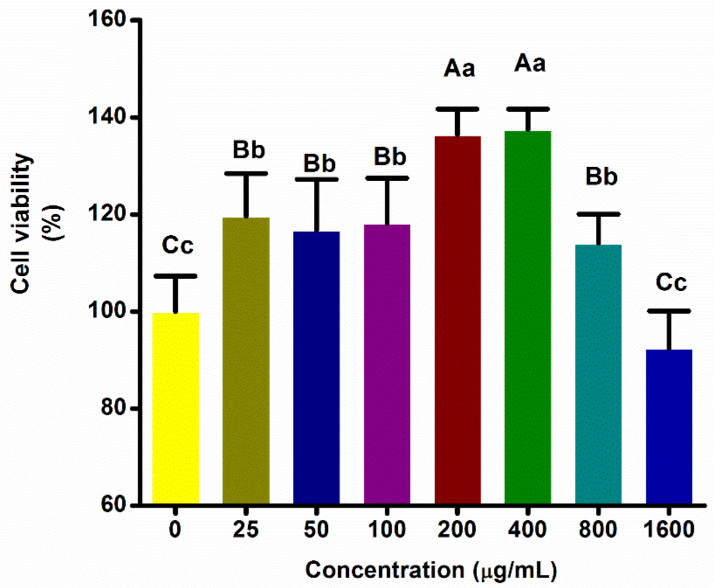

Fig. S2 The cytotoxicity of IPA on PC12 cells under normoxia.

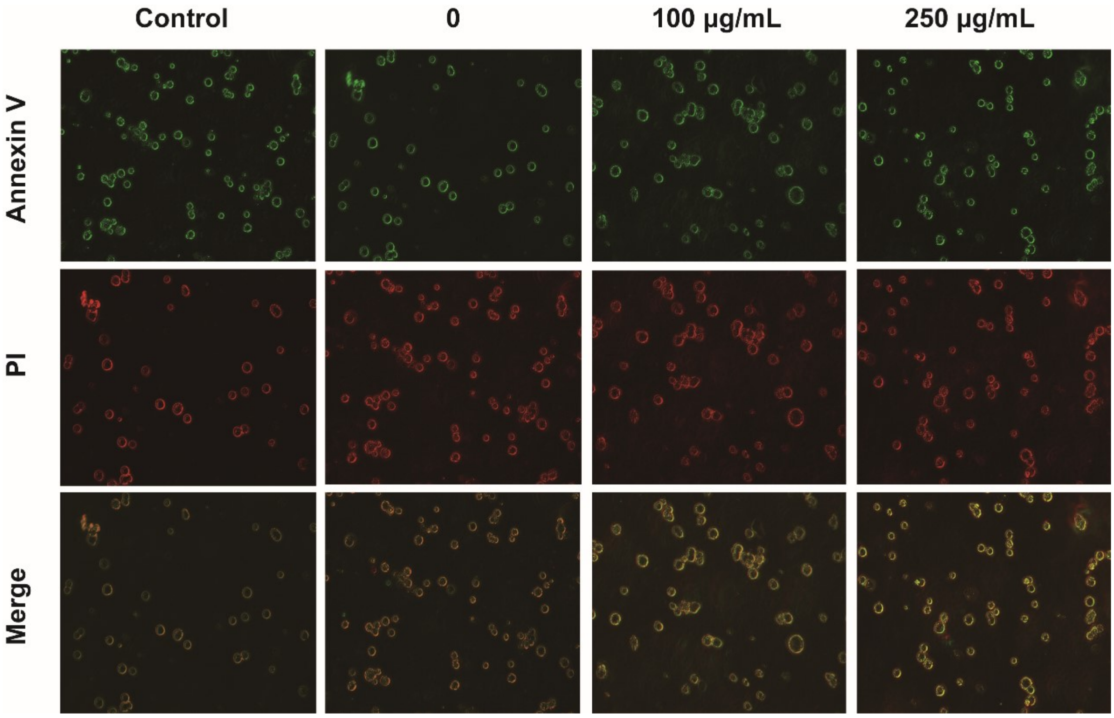

Fig. S3. Representative images showing the protective effects of IPA on cell apoptosis using Annexin-FITC and Propidium Iodide (PI) staining.
