# Supplementary material for: Phenolic Acids-Rich Fractions from Agaricus bitorguis (Quél.) Sacc. Chaidam ZJU-CDMA-12 Mycelia Modulate Hypoxic Stress on Hypoxia-Damaged PC12 Cells
Source: Molecules. 2020 Oct 21;25(20):4845. doi: 10.3390/molecules25204845 (PMC7587959; doi:10.3390/molecules25204845)
Supplement: Supplementary file 1 [file molecules-25-04845-s001.zip › supplementary files/Table S1 Data information of base peak chromatogram of IPA.pdf]

Supplementary files

Table S1 Data information of base peak chromatogram of IPA

Index	Time (min)	Area (%)	Start (min)	End (min)	Width (min)
1	0.415	6.272	0.315	0.518	0.203
2	0.550	0.326	0.518	0.648	0.130
3	0.904	0.471	0.682	0.980	0.298
4	1.015	0.217	0.980	1.103	0.124
5	1.157	0.157	1.103	1.208	0.104
6	1.352	0.749	1.208	1.394	0.186
7	1.432	0.529	1.394	1.461	0.067
8	1.490	1.022	1.461	1.723	0.262
9	1.741	0.020	1.723	1.773	0.050
10	1.886	0.126	1.807	1.914	0.106
11	2.008	0.202	1.914	2.056	0.142
12	2.243	0.758	2.056	2.372	0.316
13	2.412	0.174	2.372	2.440	0.068
14	2.472	0.221	2.440	2.509	0.069
15	2.562	0.560	2.509	2.769	0.260
16	2.837	0.164	2.769	2.909	0.140
17	3.109	0.620	2.909	3.312	0.402
18	3.335	0.060	3.312	3.382	0.071
19	3.443	0.026	3.417	3.488	0.071
20	3.506	0.009	3.488	3.523	0.035
21	3.629	0.011	3.612	3.647	0.035
22	3.718	0.024	3.647	3.736	0.089
23	3.753	0.005	3.736	3.771	0.035
24	3.797	0.013	3.771	3.824	0.053
25	3.872	0.095	3.824	3.909	0.085
26	3.925	0.007	3.909	3.942	0.033
27	4.130	0.254	4.047	4.214	0.167
28	4.230	0.016	4.214	4.247	0.033
29	4.425	0.086	4.369	4.471	0.102
30	4.487	0.009	4.471	4.503	0.033
31	4.570	0.006	4.553	4.587	0.034
32	4.623	0.039	4.587	4.689	0.102
33	4.746	0.011	4.722	4.773	0.050
34	4.941	1.388	4.823	5.228	0.405
35	5.377	0.018	5.346	5.398	0.052
36	5.483	0.081	5.398	5.552	0.155
37	5.707	0.321	5.620	5.835	0.215
38	5.958	0.250	5.886	6.035	0.149
39	6.157	0.068	6.100	6.217	0.117

40	6.282	0.148	6.217	6.332	0.115
41	6.421	0.206	6.332	6.479	0.148
42	6.550	0.074	6.479	6.595	0.116
43	6.642	0.773	6.595	6.757	0.161
44	6.868	0.624	6.757	6.984	0.228
45	7.049	0.476	6.984	7.130	0.146
46	7.344	0.305	7.295	7.408	0.114
47	7.584	5.712	7.408	8.033	0.625
48	8.103	1.263	8.033	8.162	0.129
49	8.235	2.092	8.162	8.307	0.145
50	8.359	2.970	8.307	8.467	0.160
51	8.578	2.447	8.467	8.692	0.225
52	8.813	1.304	8.692	8.950	0.258
53	9.020	1.023	8.950	9.078	0.128
54	9.182	1.471	9.078	9.336	0.257
55	9.378	0.157	9.336	9.433	0.097
56	9.480	0.357	9.433	9.546	0.113
57	9.684	2.387	9.594	9.803	0.209
58	9.825	0.019	9.803	9.852	0.048
59	10.088	5.394	9.900	10.252	0.352
60	10.319	2.556	10.252	10.443	0.191
61	10.491	0.037	10.443	10.507	0.064
62	10.713	2.628	10.507	10.875	0.368
63	10.969	0.927	10.875	11.004	0.129
64	11.074	1.808	11.004	11.180	0.176
65	11.348	3.997	11.180	11.420	0.240
66	11.532	11.199	11.420	11.998	0.578
67	12.045	0.818	11.998	12.158	0.160
68	12.244	0.474	12.158	12.286	0.128
69	12.355	0.764	12.286	12.462	0.176
70	12.525	0.950	12.462	12.623	0.161
71	12.669	0.101	12.623	12.720	0.097
72	12.896	1.784	12.720	13.137	0.417
73	13.194	0.027	13.169	13.218	0.048
74	13.477	0.732	13.362	13.683	0.321
75	13.735	0.203	13.683	13.797	0.113
76	14.065	1.897	13.829	14.231	0.402
77	14.295	0.572	14.231	14.359	0.128
78	14.392	0.346	14.359	14.423	0.064
79	14.462	0.614	14.423	14.584	0.160
80	14.642	0.248	14.584	14.680	0.097
81	14.731	0.282	14.680	14.825	0.145
82	14.920	2.182	14.825	15.118	0.292
83	15.201	1.099	15.118	15.326	0.208

84	15.554	0.206	15.488	15.618	0.130
85	15.830	0.041	15.798	15.879	0.082
86	16.083	6.989	15.944	16.431	0.487
87	16.509	0.129	16.431	16.579	0.147
88	16.953	0.056	16.904	17.002	0.097
89	17.082	0.028	17.034	17.099	0.065
90	17.186	1.569	17.099	17.308	0.209
91	17.435	2.040	17.308	17.629	0.321
92	18.089	0.044	18.052	18.117	0.065
93	18.923	0.391	18.804	19.066	0.262
94	20.690	1.324	20.558	20.890	0.331
95	21.211	0.681	21.102	21.360	0.258
96	21.856	0.141	21.790	21.968	0.178
97	23.751	1.370	23.604	23.799	0.195
98	23.860	1.081	23.799	23.934	0.135
99	24.030	1.607	23.934	24.210	0.276
100	24.252	0.283	24.210	24.297	0.087
101	24.366	0.782	24.297	24.785	0.488
